# Carpal bone replacement using personalized 3D printed tantalum prosthesis

**DOI:** 10.3389/fbioe.2023.1234052

**Published:** 2023-10-30

**Authors:** Changgui Zhang, Hao Chen, Huaquan Fan, Ran Xiong, Rui He, Chengjun Huang, Yang Peng, Pengfei Yang, Guangxing Chen, Fuyou Wang, Liu Yang

**Affiliations:** Center for Joint Surgery, Southwest Hospital, Army Medical University, Chongqing, China

**Keywords:** 3D printing, tantalum metal, carpal bone replacement, personalized, wrist

## Abstract

**Objective:** Scaphoid and lunate fractures have a relatively high incidence rate. Traditional carpectomy and carpal arthrodesis in the treatment of carpal osteonecrosis will lead to many complications. Three-dimensional (3D) printed tantalum has good biocompatibility and can be designed to match the patient’s personalized anatomical carpal structure. This study aims to investigate carpal function and prosthesis-related conditions after carpal bone replacement using 3D printed tantalum prostheses.

**Methods:** From July 2020 to January 2022 at our center, seven patients with osteonecrosis of the carpus received carpal bone replacement using 3D printed tantalum prosthesis. The Disability of the Arm, Shoulder and Hand (DASH) score and patient satisfaction, as well as the Mayo Wrist Scores (Cooney method, modified Green, and O’Brien wrist score), were used to evaluate the preoperative and postoperative wrist function of patients. The Visual Analog Scale (VAS) pain scores were also recorded before and after surgery. The angles of flexion, dorsiflexion, ulnar deviation, and radial deviation were measured using an arthrometer. The grip strength and pinch strength of the operated hand after carpal bone replacement and the contralateral healthy carpus were measured using a dynamometer. Radiographs were taken to confirm the condition and complications of the tantalum prosthesis.

**Results:** All seven patients were followed for 19.6 ± 2.7 months. At the last follow-up, the grip strength of the operated wrist joint after carpal bone replacement was 33.4 ± 2.3 kg, the pinch strength was 8.9 ± 0.7 kg, the flexion was 54.6° ± 0.8°, the dorsiflexion was 54.7° ± 1.7°, the ulnar deviation was 34.6° ± 1.9°, and the radial deviation was 25.9° ± 0.8°, all of which showed no statistically significant difference with the contralateral healthy carpus (*p* > 0.05). There were significant differences in the VAS, DASH, and MAYO scores between the preoperative and the last follow-up (*p* < 0.01). Patients had reduced postoperative pain and improved wrist function and range of motion (ROM), and the tantalum prostheses were stable.

**Conclusion:** The 3D printed tantalum brings us new hope, not only for hip or knee replacement, but also for joint replacement of other complex anatomical structures, and patients with other irregular bone defects such as bone tumors and deformity, which could realize personalized treatment and precise medicine.

## Introduction

Scaphoid and lunate fractures are quite common and account for 2% of total body fractures; scaphoid fractures account for 60%–70% of carpal fractures, and people who develop such fractures are mostly young adults (Borges et al., 2020). Due to the personalized anatomical structure, scaphoid and lunate fractures have a very high malunion rate. They may even lead to bone nonunion, osteonecrosis, and resorption collapse, resulting in pathological changes of the scapholunate joint after trauma (Penteado et al., 2012; Camus and Van Overstraeten, 2022). Many treatment options are used to treat scapholunate necrosis, including necrotic bone resection, vascularized tendon and bone replacement, scapho-trapezio-trapezoid fusion (STTF), proximal row carpectomy, and total carpal arthrodesis. However, patients with stage III-IV lunate osteonecrosis, STTF, proximal row carpectomy, and total wrist arthrodesis experience a greater sacrifice in carpal joint function (Fortin and Louis, 1993; Sauerbier et al., 2000; Meier et al., 2004; De Smet et al., 2005; Yasuda et al., 2005; Croog and Stern, 2008; Lumsden et al., 2008). This is also the reason why conventional surgery could not achieve satisfactory results. The lunate and scaphoid, combined with many ligaments of the carpus, trapezium, trapezoid, and distal radius, form the carpal joint, which is very important for the stability and force transmission of the carpus (Langer et al., 2019; Artuso et al., 2022). This urges us to find the best possible way to preserve the carpal bone or restore the anatomy of the carpus.

The emergence of artificial lunate prosthesis aims to preserve the normal intercarpal anatomy and maximize the function of the carpal joint. Artificial prosthesis replacement of avascular necrotic lunate is of great significance to maintaining the anatomical relationship and stability of each carpal bone and improving carpal joint function. The first generation of prosthetic material was silicone, but it was gradually disused due to its high incidence of prosthetic rupture and silicone synovitis (Swanson, 1970; Kato et al., 1986; Kaarela et al., 1998; Schuind et al., 2008). Many prosthetic materials have been applied in scaphoid and lunate replacement, such as silicone rubber, cobalt chromium molybdenum alloy, acrylic, cobalt chromium, and titanium; however, these materials may lead to synovitis, arthritis, stress shielding, and other complications. Finding the most suitable prosthetic material has always been a challenge for us (Lippman and McDermott, 1949; Agerholm and Goodfellow, 1963; Barber and Goodfellow, 1974; Marcuzzi et al., 2011; Parwaiz and Elnikety, 2018).

Porous tantalum is currently recognized by the medical community as the most biocompatible metal implant material. It has high porosity, low modulus of elasticity, high coefficient of friction, tissue endogeneity, and cartilage conductivity, and its primary and long-term stability in clinical application have been verified; however, traditional manufacturing methods cannot achieve the fabrication of personalized porous tantalum implant prostheses (Swanson, 1970; Shimko et al., 2005; Fan et al., 2021). The lunate, as a bridge for carpal joint movement, has an irregular shape, and its anatomical structure is complex. On the other hand, the anatomical shapes of the carpal bones vary greatly, and the commercialized carpal prosthesis cannot match the complex anatomical structure. Personalized three-dimensional (3D) printing technology can print the prosthesis completely according to the anatomical structure of the patient’s carpus. The 3D printed carpal prosthesis can not only restore the original anatomy of the capitate-lunate joint and radio-lunate joint but also realize the tight union among the proximal row of carpal bones to maximize the function and stability of the carpal joint (Kanagasuntheram et al., 2019). In this study, we performed lunate and scaphoid bone replacement with 3D printed tantalum, which we believed was the first attempt in the world. During the follow-ups, we found that patients had great improvement in grip strength and wrist movement after surgery, indicating that tantalum has good biocompatibility (Jiang et al., 2023).

## Treatment process

### Materials and methods

Patients with carpal fractures treated at our center from July 2020 to January 2022 were followed up. Surgical indications were as follows: 1. Patients who received 6 months of conservative treatment but still had symptoms; 2. Patients with stage III-IV lunate and scaphoid osteonecrosis; 3. Patients unable to work due to carpal symptoms; 4. Patients unwilling to continue to conservative treatment. Inclusion criteria were as follows: 1. Patients who underwent 3D printed tantalum carpal bone replacement due to carpal osteonecrosis; 2. Preoperative imaging data were complete. Exclusion criteria were as follows: 1. Patients with severe cardiovascular disease and other systemic diseases; 2. Local or distal active infection of the carpal joint; 3. Mental illness. Finally, seven patients were obtained: there were four cases of lunate replacement and three cases of scaphoid replacement, six were males and one was female, and the average age was 29.0 years (17–58 years). All patients underwent 3D printed tantalum carpal bone replacement.

All patients were informed before surgery of the risk of using 3D printed tantalum for carpal bone replacement, and second-stage surgery might be required. All surgeries were performed by the same senior surgeon. This study was approved by the hospital’s Ethics Committee (No. KY2023024). All patients signed the informed consent and agreed to be included in the study.

### Preoperative application of personalized 3D printing technology

**FIGURE 1:**
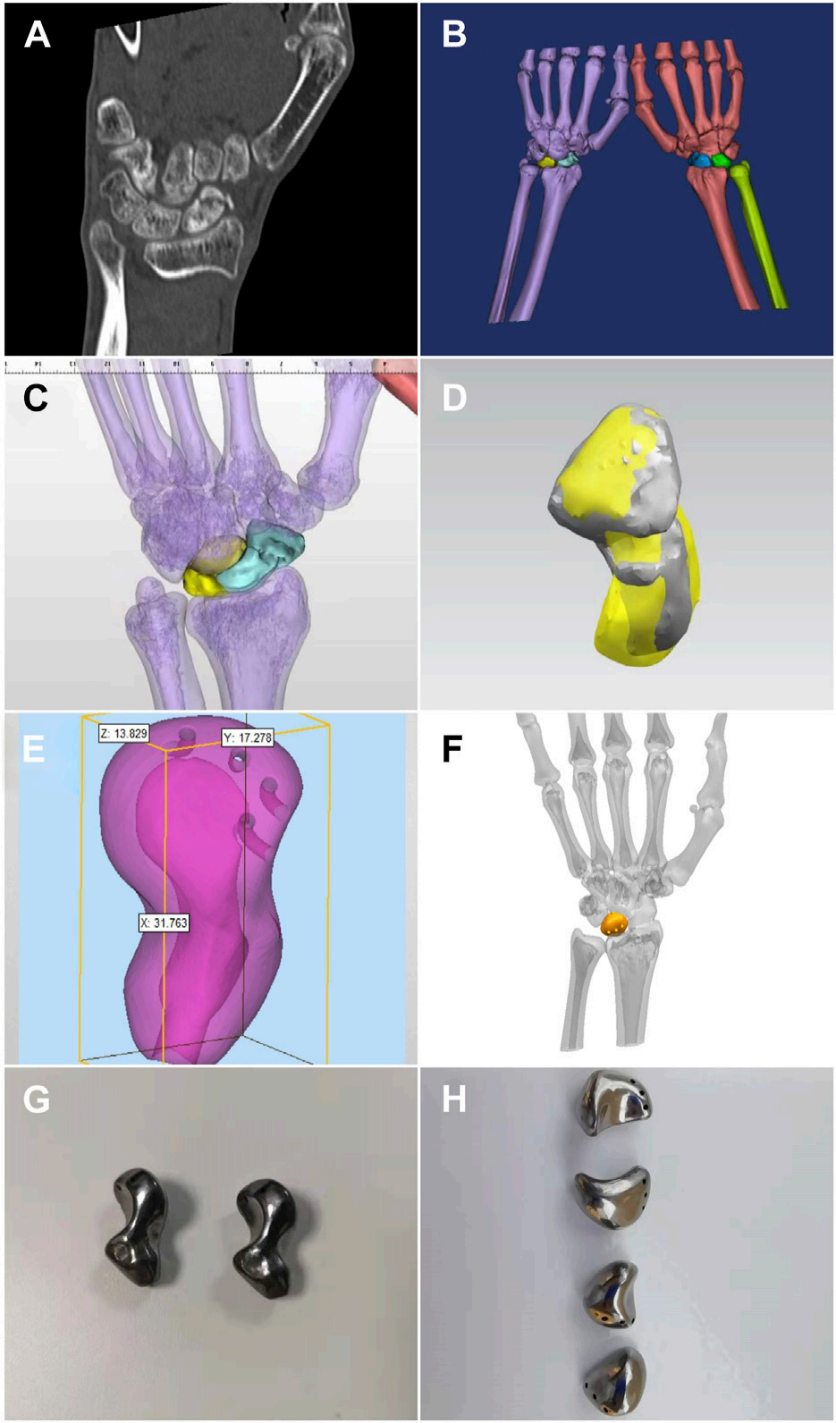
Carpal prosthesis design process. **(A)** Preoperative CT thin-layer scan; **(B)** Bilateral carpal 3D model reconstruction; **(C)** Mirror image of the healthy scaphoid coincides with the affected side after flipping; **(D)** Frontal view of the 3D model of the scaphoid prosthesis design; **(E)** Size measurement of the scaphoid prosthesis; **(F)** Frontal view of the 3D model of the lunate prosthesis design; **(G)** 3D printed tantalum scaphoid prosthesis; **(H)** 3D printed tantalum lunate prosthesis.

A 1:1 isometric lesion model was prepared before surgery, and a tantalum lunate or scaphoid prosthesis was fabricated according to the anatomical structure of the specific patient. A 3D computed tomography (CT) thin-slice scan (slice thickness, 1.0 mm; Siemens, Germany) of the carpus was performed. The DICM data were extracted and imported into MIMICS software to reconstruct the 3D data of the lesion and surrounding tissues; the reconstructed data were imported into SIEMENS NX 3D design software to convert the designed data into STL format and import it into a 3D printer (UP BOX, Beijing Tiertime Technology Co., Ltd., China). For raw material, polylactic acid polymer (PLA, Beijing Tiertime Technology Co., Ltd., China) was used for preparation of the personalized lesion models. The anatomical shape and the shape of the adjacent carpal articular surface were used to design the prosthesis individually. If the lunate and scaphoid bones were seriously damaged and there were obvious deformities, the carpal bone prosthesis on the affected side could be designed by flipping the healthy carpal bone using mirror image technology. The structure of the prosthesis was set according to the bony condition of the carpus. The appearance of the carpal prosthesis was highly polished, and the framework was a tantalum structure with a thickness of 3.5–4 mm. The prosthesis was a hollow structure, and three arc-shaped tunnels with a diameter of 1.5 mm were designed on the back side for suture fixation. The tantalum carpal prosthesis was printed after the design was completed (Figure 1). During the preparation of the lesion model, different colors were used to distinguish the lesion and the surrounding healthy tissue. This could help improve the surgical rehearsal and surgical plan. The prepared model and porous tantalum carpal prosthesis were sealed and packaged and sterilized with ethylene oxide for use in the surgery.

### Surgical techniques

**FIGURE 2:**
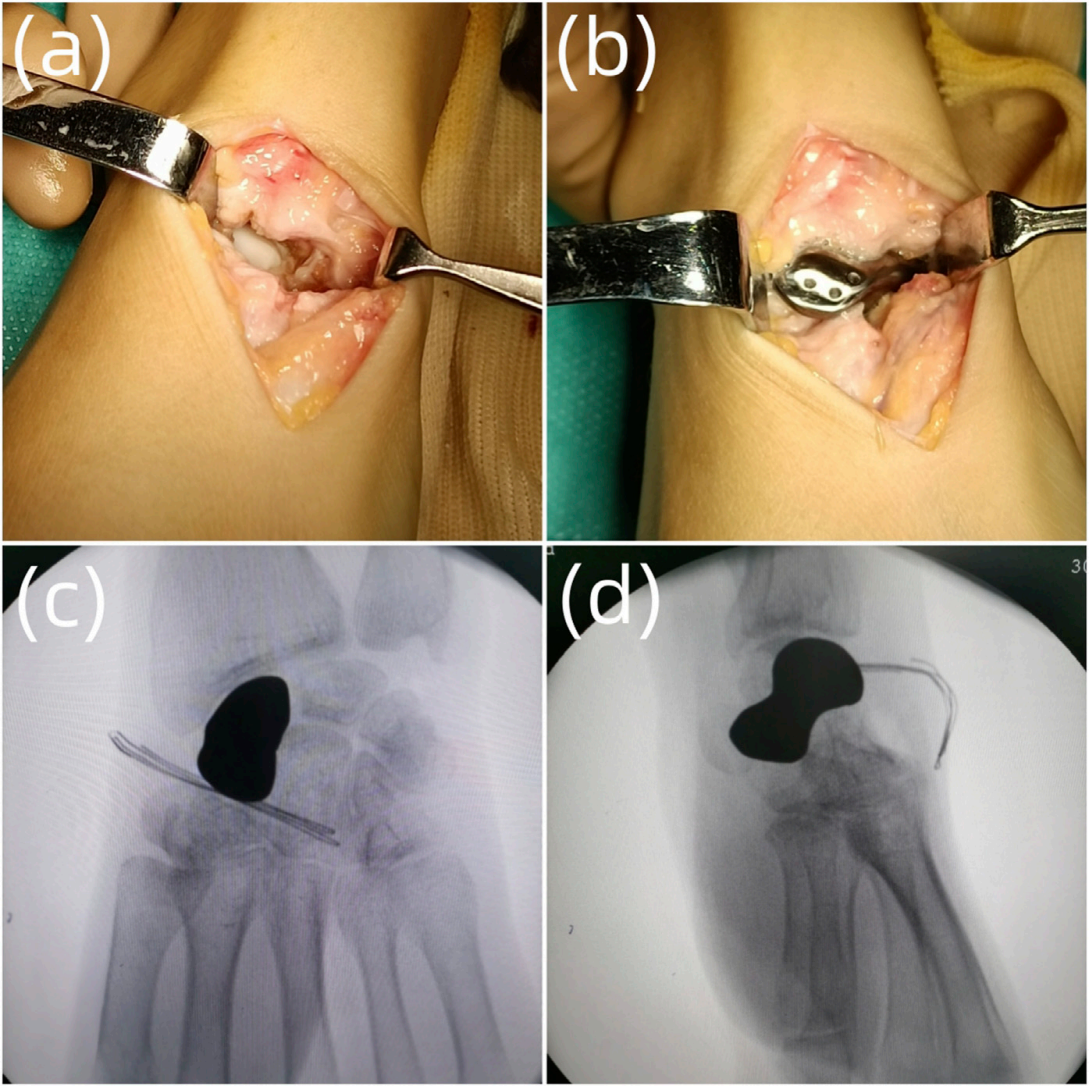
The surgical process. **(A)** Debridement of the scaphoid necrotic tissue; **(B)** Implantation of the scaphoid prosthesis; **(C)** Intraoperative anteroposterior radiograph of the scaphoid prosthesis after implantation; **(D)** Intraoperative lateral radiograph of the scaphoid prosthesis after implantation.

**FIGURE 3:**
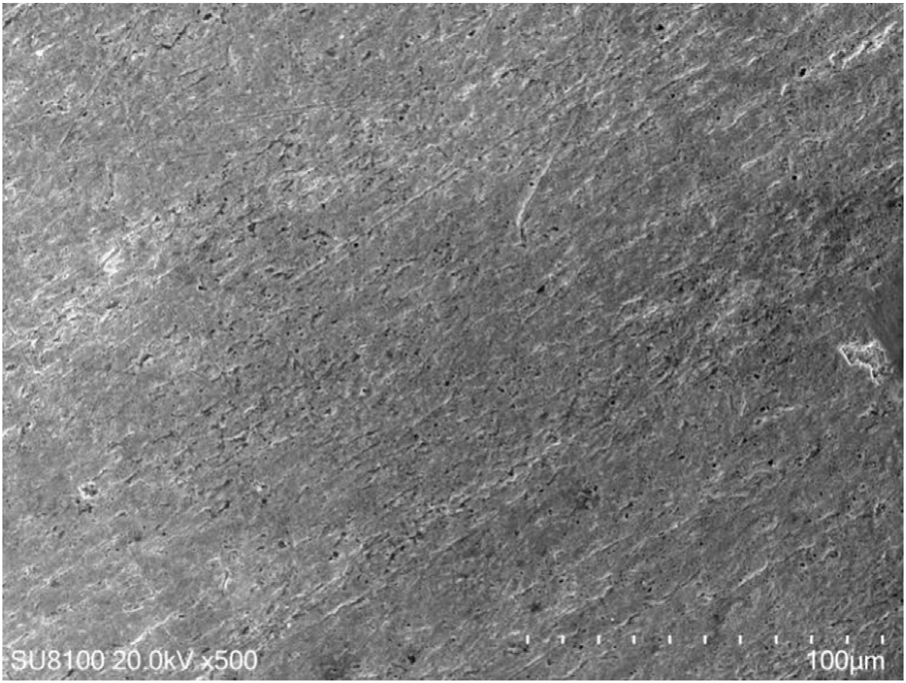
Tantalum metal structure under electron microscope.

After successful anesthesia, C-arm fluoroscopy was applied to locate the proximal end, distal end and the axis of scaphoid, and mark the surgical incision. Routine surgery preparations were conducted. Sterile tourniquet and draping were applied intraoperatively, and the tourniquet pressure was set at 220 mmHg. The dorsal approach was chosen to incise the skin, subcutaneous tissue, and fascia and expose and retract the tendon of the extensor pollicis longus. A T-shaped capsulotomy was conducted to expose the scaphoid. Collapse of scaphoid and cartilage degeneration were observed; the proximal and distal end and surrounding tissue ligaments were relatively complete. The scaphoid and surrounding adhesion tissues were separated, the necrotic scaphoid was completely resected, the articular surface and the hyperplastic synovium around it were debrided, and the wound was rinsed with normal saline. The tantalum prosthesis was implanted into the scaphoid, and we tested its stability and whether impingement existed. The prosthesis was sutured with the surrounding tissue through the tunnels at the back. The wrist was moved, and C-arm fluoroscopy was used to confirm whether the prosthesis was in a good position and whether there was impingement with passive wrist joint movement. The incision was sutured, and the surgery was complete. The intraoperative blood loss and tourniquet time were recorded (Figures 2, 3).

### Postoperative treatment

Patients were advised to move the metacarpophalangeal joints appropriately; vancomycin was applied to prevent infection; analgesia and symptomatic treatment was given; regular incision dressing changes were conducted after surgery; the leakage of wound dressings was observed. Anteroposterior and lateral radiographs of the wrist joints were checked during the follow-ups. If the incisions healed normally and the prosthesis was well positioned, patients were instructed to move the operated limb in four directions, including carpal flexion, dorsiflexion, ulnar deviation, and radial deviation, to improve joint function and avoid postoperative thrombosis.

### Outcome indicators

Preoperative and postoperative visual analog scale (VAS) scores, disability of the arm, shoulder and hand (DASH) scores and patient satisfaction, and Mayo wrist scores (Cooney method, modified Green and O’Brien wrist score) of the patients were recorded. The angles of flexion, dorsiflexion, ulnar deviation, and radial deviation of the carpus were measured using the arthrometer. Exacta™ (B&L Engineering, United States) was used to measure hand grip strength and pinch strength of the operated limb after carpal bone replacement and the contralateral healthy carpus. All patients were followed up for 19.6 ± 2.7 months (12–29 months). At the last follow-up, radiographs were required to confirm the condition and complications of the tantalum prosthesis.

### Statistical analysis

Analysis was carried out by SPSS 22.0 statistical software (SPSS; Chicago, IL, United States). The count data were expressed as mean ± standard deviation. If conformed to normal distribution, a paired *t*-test was used, and *p* < 0.05 was considered statistically significant.

## Results

### Follow-up results

All seven patients were followed up for 19.6 ± 2.7 months (12–29 months). At the last follow-up, the grip strength of the contralateral healthy carpal joint was 35.1 ± 3.1 kg, the pinch strength was 9.5 ± 0.7 kg, and the flexion was 56.6° ± 1.1°, dorsiflexion 58.3° ± 0.6°, ulnar deviation 36.9° ± 1.2°, radial deviation 28.3° ± 0.6°; grip strength of the operated side at the last follow-up was 33.4 ± 2.3 kg, pinch strength 8.9 ± 0.7 kg, and the flexion was 54.6° ± 0.8°, dorsiflexion was 54.7° ± 1.7°, ulnar deviation was 34.6° ± 1.9°, radial deviation was 25.9° ± 0.8°, and there was no statistical difference compared with the contralateral healthy carpus (*p* > 0.05) (Table 1). The preoperative VAS score was 6.9 ± 0.7, and at the last follow-up, the VAS score was 0.3 ± 0.2, which was significantly reduced (*p* < 0.01). The preoperative DASH score was 38.4 ± 0.6, and at the last follow-up it was 6.8 ± 0.4; the preoperative MAYO score was 7.3 ± 0.5, and the last follow-up it was 80.9 ± 1.2, both showing significant statistical differences compared with preoperative scores (*p* < 0.01) (Table 2). At the last follow-up, no complications such as osteoarthritis were found in the patients’ radiographic examinations.

**TABLE 1:** The carpus receiving carpal bone replacement and the contralateral healthy carpus.

Follow-up indicators	Carpal bone replacement group	Contralateral healthy carpus	*p*-Value
Grip strength	33.4 ± 2.3	35.1 ± 3.1	0.172
Pinch force	8.9 ± 0.7	9.5 ± 0.7	0.150
Flexion	54.6 ± 0.8	56.6 ± 1.1	0.080
Dorsiflexion	54.7 ± 1.7	58.3 ± 0.6	0.073
Ulnar deviation	34.6 ± 1.9	36.9 ± 1.2	0.066
Radial deviation	25.9 ± 0.8	28.3 ± 0.6	0.055

**TABLE 2:** Data at the last follow-up of carpal bone replacement.

Follow-up indicators	Preoperative	Postoperative	*p*-Value
VAS	6.9 ± 0.7	0.3 ± 0.2	0.0001
MAYO	7.3 ± 0.5	80.9 ± 1.2	0.0001 0.0001
DASH	38.4 ± 0.6	6.8 ± 0.4	

MAYO, Mayo wrist score; VAS, visual analog scale; DASH, disability of the arm, shoulder and hand.

### Typical case

**FIGURE 4:**
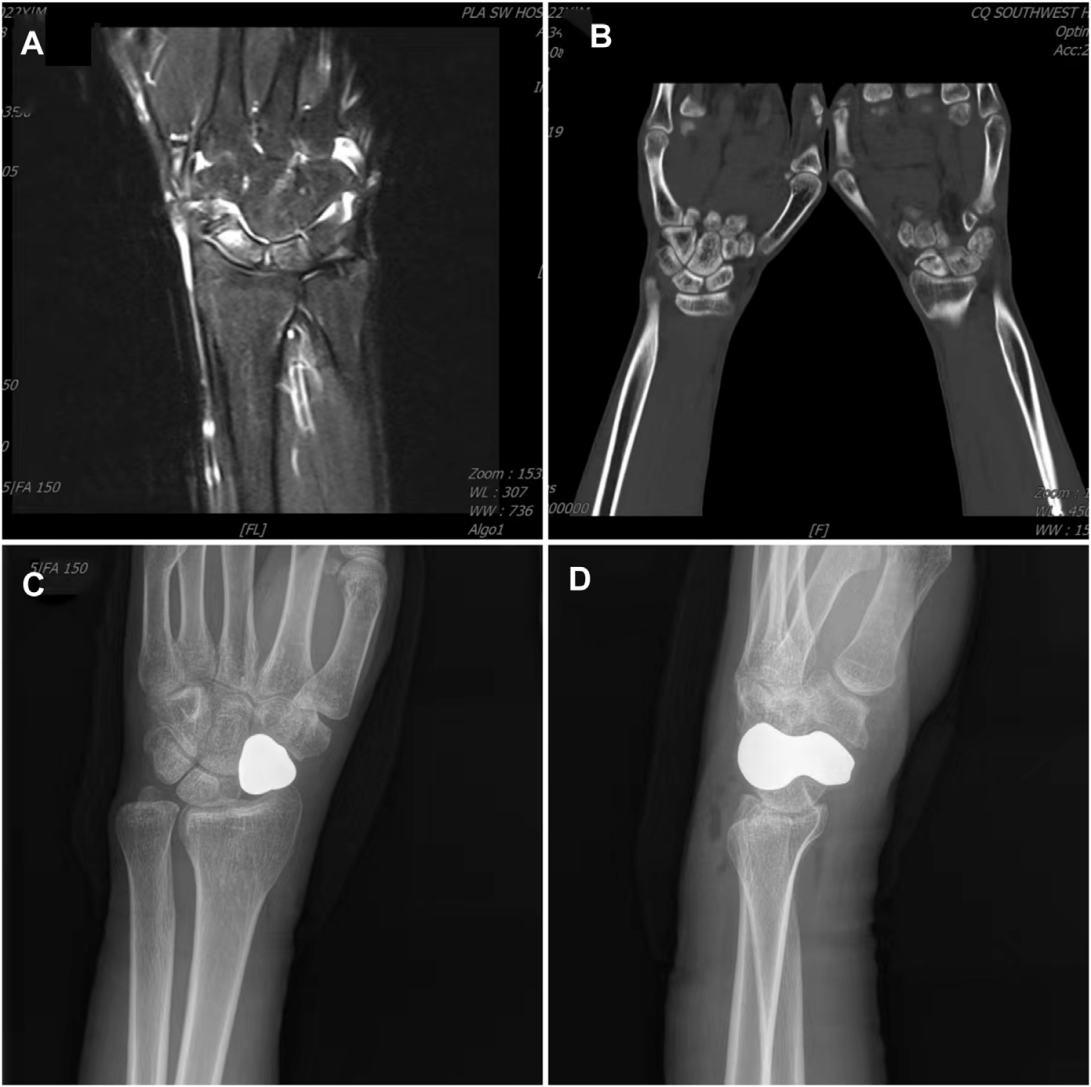
Typical case. **(A)** Preoperative MRI shows scaphoid osteonecrosis; **(B)** Preoperative CT also shows scaphoid osteonecrosis; **(C)** Anteroposterior radiograph of the carpal joint 2 days after surgery; **(D)** Lateral radiograph of the carpal joint 2 days after surgery.

**FIGURE 5:**
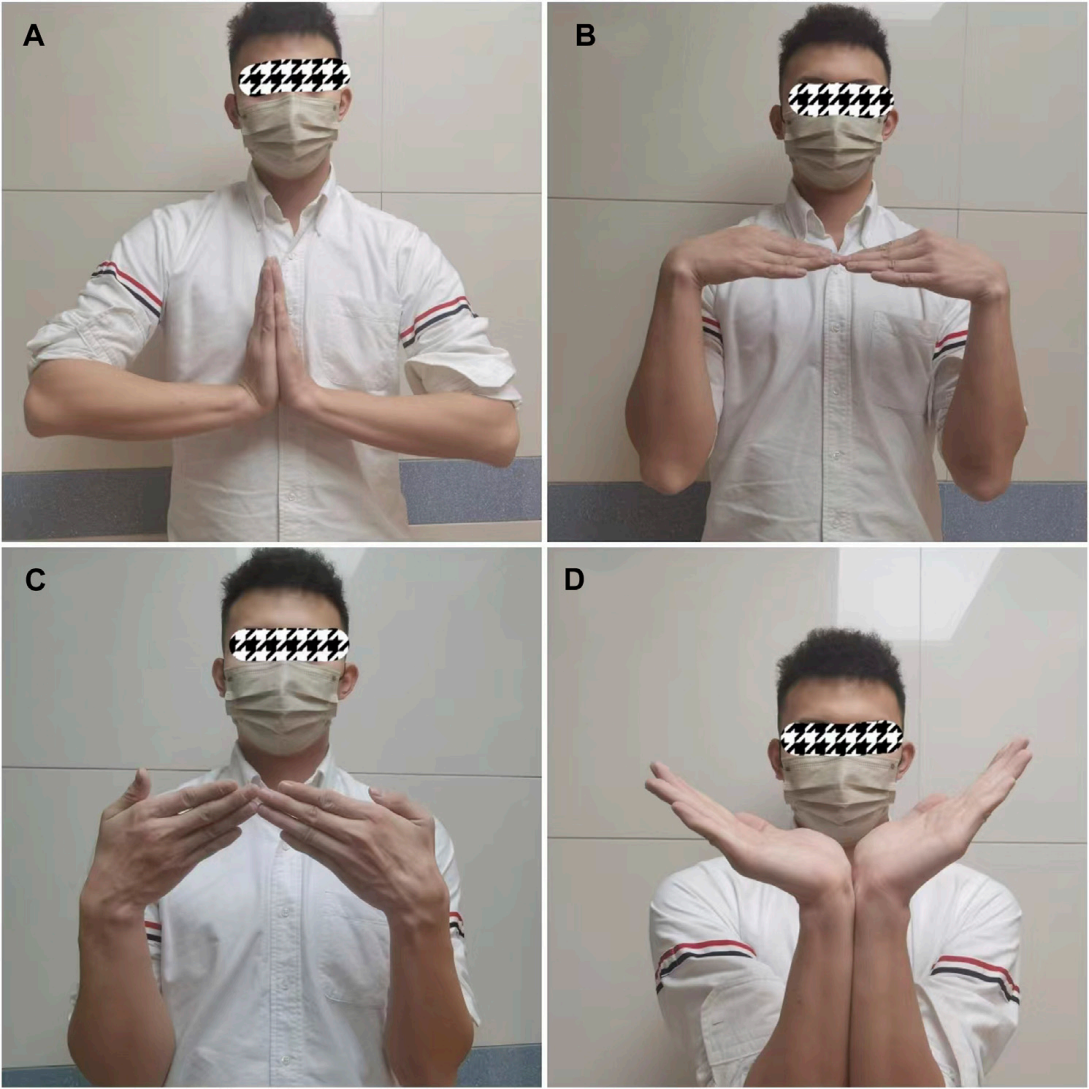
Typical case. **(A)** Dorsiflexion function of the carpus 20 months after surgery; **(B)** Flexion function of the carpus 20 months after surgery; **(C)** Ulnar deviation function of the carpus 20 months after surgery; **(D)** ROM of carpal dorsiflexion 20 months after surgery.

**FIGURE 6:**
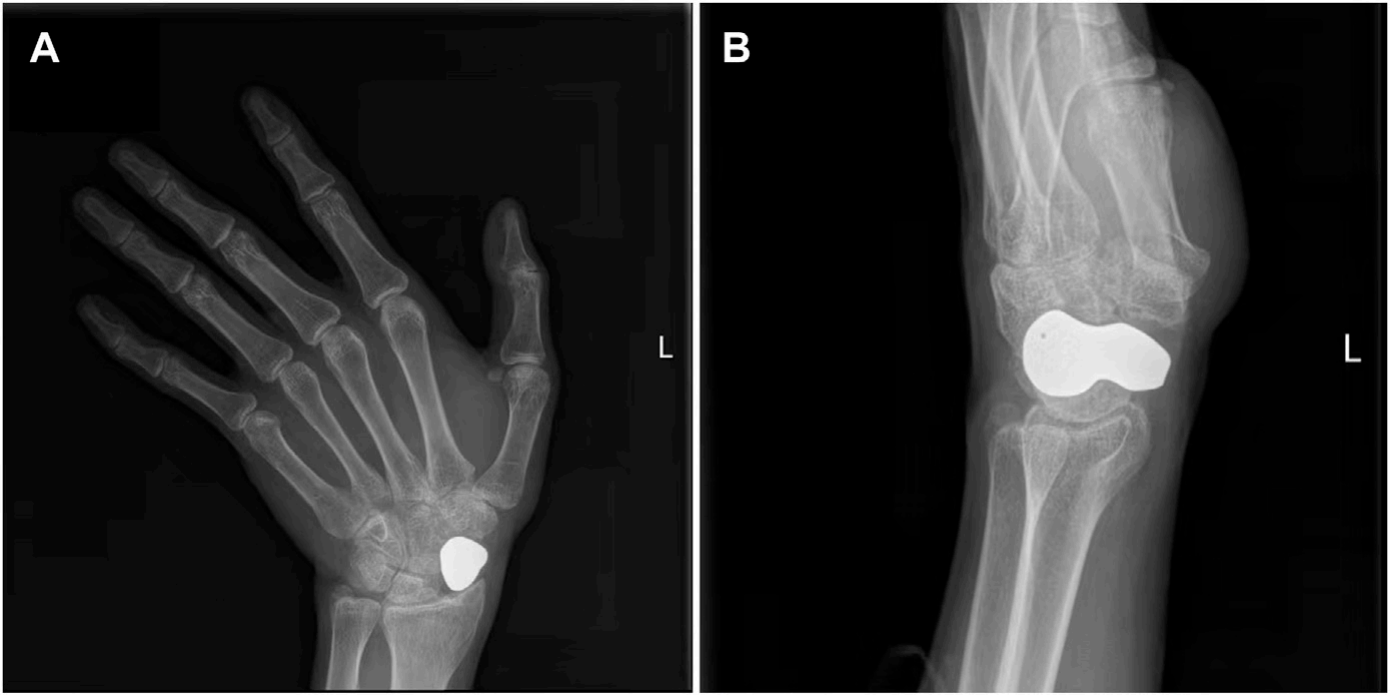
Typical case. **(A)** Anteroposterior radiograph of the scaphoid prosthesis 20 months after surgery; **(B)** Lateral radiograph of the scaphoid prosthesis 20 months after surgery.

The patient was admitted to the hospital due to left carpal pain and limited mobility for 2 years. The patient suffered left carpal joint swelling and pain with limited mobility due to a fall while exercising 2 years ago. Conservative treatment was conducted for half a year, but the condition did not improve. Magnetic resonance imaging (MRI) examination revealed an old fracture of the left scaphoid with necrosis. Physical examination showed mild swelling of the left carpal joint, obvious local tenderness on the radial and back side of the left carpal joint, and limited range of motion (ROM) of flexion, dorsiflexion, ulnar deviation, and radial deviation. At the 20-month follow-up appointment, the patient had no pain in the left carpal joint, the ROM of the left carpal joint had recovered satisfactorily, and the function was significantly improved. The grip strength was 34 kg, which was no different from that of the healthy side. The DASH score was 7, the MAYO wrist joint score was 79, and the tantalum prosthesis was stable without dislocation (Figures 4–6).

## Discussion

Avascular necrosis of the scaphoid and lunate can cause severe pain and limited carpal movement, and it may lead to carpal collapse and osteoarthritis. Relief of pain, preservation of carpal motion, maintenance of carpal strength, and good function are the keys to the treatment of carpal osteonecrosis. Many treatment options are used to treat necrosis of the scapholunate bone, including necrotic bone resection, vascularized tendon and bone replacement, STTF, carpectomy, and total carpal arthrodesis. Bone grafting has a risk of infection, and carpectomy and total carpal arthrodesis have a great impact on carpal joint function.

The preferred treatment for stage III-IV lunate and scaphoid osteonecrosis is carpal bone replacement. In 1970, Swanson. (1970) completed the first case of lunate replacement with a silicone prosthesis and later found that the silicone prosthesis could cause synovitis. Early artificial materials for carpal prostheses also included tendon prostheses, artificial ceramics, dental base acrylic resin powder, etc., Due to their poor stability, they are prone to cartilage wear and reactive inflammation in the long term and are no longer used in clinical practice. Kanatani et al. (2010) reported two cases of replacement of necrotic lunate with artificial silicone rubber. Both patients had wrist degeneration and carpal tunnel syndrome. Viljakka et al. (2014) reported the long-term follow-up of 53 cases where artificial silicone rubber had been used to replace necrotic lunate and confirmed that silicone rubber was not a suitable material for lunate replacement. At present, bone cement is mostly used in the fabrication of artificial lunate prostheses, but it is not easy to prepare the bone cement prosthesis corresponding to the articular surface and the shape of the original lunate bone. The appearance of the bone cement prosthesis is hard and rough, and chronic degeneration often occurs after surgery. In addition, the elasticity modulus of non-metallic material is significantly different from that of normal bone, which has a significant impact on stress conduction, so metal carpal prostheses are the first choice for replacement.

Nowadays, titanium and its alloys are the relatively mature bone filling materials in clinical application, but studies have found that long-term complications such as loosening and stress shielding of the prosthesis are prone to occur in the long-term follow-up after titanium prosthesis implantation (Parwaiz and Elnikety, 2018). In addition, the osseointegration ability and antibacterial properties of titanium alloy materials are still far from achieving the expected effects clinically. A variety of structural and coating property variation technologies for titanium alloys have been proposed, many of which are still in the experimental research stage.

Porous tantalum has a honeycomb-like 3D structure similar to human cancellous bone, with features of low elastic modulus and high coefficient of friction, which is very conducive to the adhesion and growth of bone cells, and its internal transportation network is also very conducive to nutrient exchange; in addition, studies have confirmed that porous tantalum has good biological safety, and most scholars recognize that a pore size of 400–600 μm and porosity of 60%–70% can meet the mechanical and biological properties requirements of a porous scaffold at the same time (Sauerbier et al., 2000; Meier et al., 2004). The relatively optimal unit cell structure is regular dodecahedron and diamond types, which have relatively better osteogenic activity and are widely applied in the field of orthopedics. Thus, porous tantalum is also called tantalum bone (Paka and Pokrowiecki, 2018; Han et al., 2019). Studies have found that the use of porous tantalum implants in arthroplasty may be associated with a reduced risk of infection (Bobyn et al., 1999; Cohen, 2002; Levine et al., 2006; Howard et al., 2011; Breer et al., 2012; Mohandas et al., 2014; Mohaddes et al., 2015; Tokarski et al., 2015), and Schildhauer et al. (2006) have found that tantalum can affect the adhesion of staphylococci and thus had antibacterial properties. In addition, porous tantalum has good osteoinductivity. A previous study (Welldon et al., 2008) suggested that porous tantalum could promote the adhesion and proliferation of osteoblasts better than pure tantalum and plastic, and the cells in the pores had a higher degree of bone differentiation.

To restore the patient’s hand function to the maximum extent, the most critical thing is to restore the anatomical structure of the patient’s carpus. The carpal joint is one of the most complex ones in the human body structure, and it is also the most flexible joint. Any change in its structure can affect the patient’s function. As a bridge for carpal joint movement, the lunate is complex and irregular in shape. In addition, the lunate and scaphoid, as the central axis of the carpal joint, play a vital role in carpal movement and force transmission. Toffoli et al. (2017) found that the shape of the lunate varied greatly from person to person. The artificial bio-prosthesis of the lunate needs to conform to the anatomical differences of individual carpal joints, but the commercialized lunate and scaphoid prostheses still cannot achieve the same effect as the original bone. This geometric mismatch will damage the ulnar cartilage and triangular fibrocartilage, resulting in arthritis of the carpus. For example, the size and strength of the tendon prosthesis and carbon fiber prosthesis cannot replace the scaphoid, which will cause the loss of patient strength and the collision between the prosthesis and surrounding tissues.

The technology of 3D printing is an ideal solution to the above problems. Compared with the previous laser printing additive manufacturing technology, powder bed electron beam additive manufacturing technology not only has the advantages in efficient manufacturing of complex structures but also outstanding features such as being clean and fast, reducing stress, etc., Porous titanium prepared by powder bed electron beam additive manufacturing technology has obtained CE, FDA, and CFDA certification and been applied in the clinic. At present, the global commercialized porous tantalum prosthesis is basically monopolized by Zimmer, United States, occupying one-third of China’s artificial joint market share. However, the preparation process used by Zimmer is traditional chemical vapor deposition technology, which cannot achieve personalized rapid manufacturing of porous tantalum prostheses, while 3D printing can fabricate carpal prostheses that perfectly match the patient’s specific anatomy. (Duan et al., 2018; Zhang et al., 2020; Zhang et al., 2022). It can produce a carpal prosthesis that perfectly fits the patient based on the patient’s specific anatomy. With the support of the National Key R&D program of China, we have overcome the high melting point of tantalum metal at 2,996°C and adopted electron beam printing technology, which has greatly improved the efficiency compared with laser printing. This enabled us to complete 3D printing within 72 h from image collection and finish the world’s first 3D printed porous tantalum radial head replacement. Follow-ups found that the functional recovery of the elbow joint was very good (Zhang et al., 2022). In this study, according to the specific anatomical structure of the patient, we designed a tantalum prosthesis that matched the patient’s carpus. The structure of the prosthesis was based on the condition of the carpal bone. The appearance of the prosthesis was fully polished, the structure was a porous tantalum metal structure, and the porosity was 75%. The hollow structure of the prosthesis was completely designed according to the elastic modulus of the carpal bone to reduce complications of stress shielding. The implanted 3D printed carpal prosthesis had the same size, structure, and shape as the original carpal bone to reconstruct the original anatomical structure of the carpus. The design of the 3D printed lunate prosthesis can realize the complete alignment of the capitate-lunate joint (concave surface of the lunate) and radio-lunate joint (convex surface of the lunate), which plays an important role in maintaining the stability of the prosthesis. This design not only takes into account the biomechanical structure of the capitate-lunate and radio-lunate articular surface but also the reconstruction of the ligaments around the lunate and scaphoid, restores its physiological and mechanical state, stabilizes the carpal bones, and improves postoperative carpal function to the greatest extent. Combining tantalum materials that are most suitable for implanting prostheses and powder bed electron beam additive manufacturing technology, we can realize personalized 3D printing tantalum carpal bone replacement, which has important innovative value and potentially huge economic and social value.

A postoperative follow-up study on the implantation of 3D printed tantalum prosthesis was conducted and found that the implanted tantalum carpal prosthesis could completely reconstruct the anatomical structure of the carpal joint, relieve pain, and restore the grip strength, pinch strength, and carpal movement of patients. During the follow-up, no complications such as arthritis, carpal pain, and cartilage damage caused by the prosthesis were found. This was because the 3D printed prosthesis completely matched the surrounding structure, and there was no impact or cartilage damage. On the other hand, the follow-up also found that the grip and pinch strength of the patients could better transmit force. We speculated that this was because the elastic modulus of tantalum was similar to that of cancellous bone, which could restore the anatomical structure of the carpus and the force of the elbow joint could be transmitted to the hand so that the patient could have better grip strength and ROM. Studies found that patients who used previous prostheses made of other materials had a decrease in grip strength and ROM. Maes-Clavier, (2011) observed that 58% of the patients had a decrease in strength at the last follow-up after scaphoid replacement using pyrocarbon prostheses. Pierrart et al. (2012) followed 11 patients with scaphoid pyrocarbon prostheses and found that only 1 patient’s grip strength recovered to the preoperative level; the rest of the patients lost their grip strength, which was only 27.42% of the uninjured side. Daruwalla et al. (2013) followed 12 patients with pyrocarbon prostheses in the scaphoid and found that the flexion and extension of the carpus were only 54% and 67% of those of the healthy side, and the radial and ulnar deviations were only 58% and 71% of those of the healthy side; the postoperative average grip strength was 65% of that of the contralateral hand, and the pinch strength was only 73% of that of the contralateral hand. This study also has some limitations. The follow-up period is relatively short. In addition, due to the small number of cases for carpal prostheses, the number of patients included in this group is also relatively small. Future multicenter case-controlled studies will overcome these limitations.

## Conclusion

The 3D printed tantalum carpal prosthesis used in this study can completely match the patient’s anatomical structure, relieve pain, restore the patient’s carpal mobility, and reduce complications. We can apply 3D printing technology to achieve personalized and precise treatment. There is some basic research on 3D printing tantalum, but it lacks the support of clinical study. This study is an important supplement to the research on tantalum metal.

## Data Availability

The original contributions presented in the study are included in the article/Supplementary Material; further inquiries can be directed to the corresponding authors.
